# Targeting gut microbiota in aging-related cardiovascular dysfunction: focus on the mechanisms

**DOI:** 10.1080/19490976.2023.2290331

**Published:** 2023-12-10

**Authors:** Siqi Liu, Yufeng He, Yali Zhang, Zhaolun Zhang, Keming Huang, Li Deng, Bin Liao, Yi Zhong, Jian Feng

**Affiliations:** a Department of Cardiology, The Affiliated Hospital of Southwest Medical University, Luzhou, Sichuan, People’s Republic of China; b Department of Rheumatology, The Affiliated Hospital of Southwest Medical University, Luzhou, Sichuan, People’s Republic of China; c Department of Cardiovascular Surgery, The Affiliated Hospital of Southwest Medical University, Luzhou, Sichuan, People’s Republic of China

**Keywords:** Gut microbiota, metabolites, aging, cardiovascular dysfunction, drug therapy

## Abstract

The global population is aging and age-related cardiovascular disease is increasing. Even after controlling for cardiovascular risk factors, readmission and mortality rates remain high. In recent years, more and more in-depth studies have found that the composition of the gut microbiota and its metabolites, such as trimethylamine N-oxide (TMAO), bile acids (BAs), and short-chain fatty acids (SCFAs), affect the occurrence and development of age-related cardiovascular diseases through a variety of molecular pathways, providing a new target for therapy. In this review, we discuss the relationship between the gut microbiota and age-related cardiovascular diseases, and propose that the gut microbiota could be a new therapeutic target for preventing and treating cardiovascular diseases.

## Introduction

Morbidity and mortality rates from age-related cardiovascular diseases such as atherosclerosis, hypertension, and heart failure remain high worldwide. Despite known risk factors (such as smoking, blood lipids, and blood sugar) and the increasing availability of new targeted drugs, age-related cardiovascular disease continues to pose a threat to global health.

**Figure 1.**
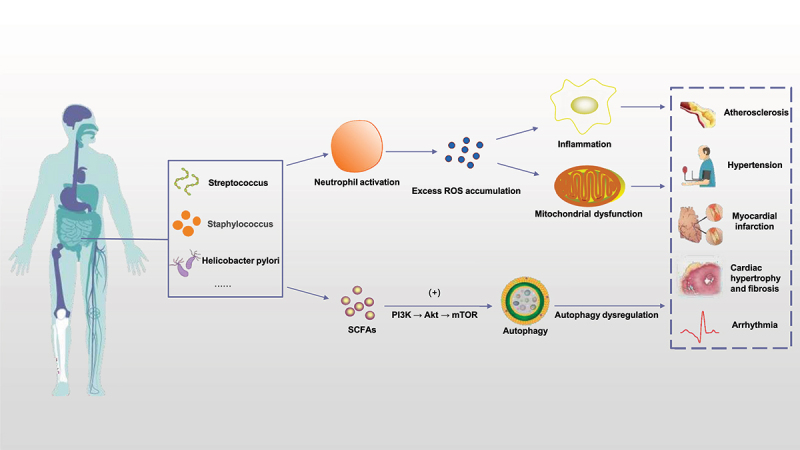
Gut microbiota and cardiovascular disease. Various gut microbiota in the human body, such as *Streptococcus, staphylococcus* and *Helicobacter pylori*, can activate neutrophils in various ways to cause inflammatory reaction, thus causing excessive ROS accumulation in the body and damaging mitochondrial function; on the other hand, SCFAs produced by gut microbiota ingesting dietary components can also trigger autophagy through PI3K/Akt/mTOR pathway. The above mechanisms play a key role in age-related cardiovascular diseases such as atherosclerosis, hypertension, myocardial infarction, myocardial hypertrophy and fibrosis. (ROS: reactive oxygen species. SCFAs: short-chain fatty acids).

A healthy gut microbiota is essential for human health. The gut microbiota composition and metabolites will change with age, dietary habits, and other factors, and are affected by external factors such as supplementation with probiotics and the use of antibiotics. More recently, fecal microbiota transplantation (FMT), microbial enzyme inhibitors, gut microbiota metabolites, and probiotics supplements have been developed as new precision-medicine approaches to modulating the gut microbiota.^1,2^ Understanding the relationship between the gut microbiota and age-related cardiovascular disease is crucial for the prevention and treatment of cardiovascular disease, as well as the development of targeted therapies for cardiovascular disease. In this review, we will discuss the pathogenesis of age-related cardiovascular disease and the role of the gut microbiota, with the aim of highlighting new directions for the treatment of age-related cardiovascular disease in the future (Figure 1).

## Potential mechanisms of cardiovascular aging

Cardiovascular system aging includes progressive dynamic changes in structure and function that can lead to impaired cardiovascular homeostasis, manifested by a reduction in cardiomyocyte numbers, hypertrophy of aging cardiomyocytes, an increased inflammatory response, progressive development of cardiac fibrosis, decreased elasticity, increased stiffness, and a series of clinical diseases.^3^ Therefore, exploring the mechanism of cardiovascular aging could help prevent some cardiovascular diseases and improve or even reverse cardiovascular function.

### Oxidative stress

Oxidative stress plays an important role in age-related cardiovascular disease. Increased reactive oxygen species (ROS) production or impaired antioxidant production are considered to be key factors in the occurrence and development of age-related cardiovascular diseases.^4,5^ Aging itself is a risk factor for cardiovascular diseases, and aging is related to oxidative stress, which can also lead to accelerated cell aging and organ dysfunction. Babcock et al. showed that endothelial dysfunction is accelerated by decreased testosterone levels, increased ROS accumulation, and weakened antioxidant defenses in middle-aged and older men, and that supplementation with the antioxidant vitamin C can mitigate these changes.^6^

Some components of the gut microbiota themselves, or their metabolites, such as TMAO and SCFAs, can regulate exogenous and endogenous ROS levels. *Lactobacillus* can enhance the permeability of gastrointestinal mucosa or increase the secretion of intestinal mucus, thereby stimulating ROS production.^7^ *Helicobacter pylori* activates NADPH oxidase to produce ROS and activates REDOX-sensitive signal transduction via NF-κB, activator protein-1 (AP-1), and mitogen activated protein kinase (MAPK) to induce the release of inflammatory cytokines.^8,9^ TMAO is a byproduct of gut microbiota metabolism of phosphatidylcholine, carnitine, betaine, and other nutrients.^10^ Increased circulating TMAO levels caused by increased choline intake may inhibit AMPK and SIRT1 expression levels, increase ROS levels, and activate inflammatory responses.^11^ Lipopolysaccharide (LPS) is a specific component of the cell wall of Gram-negative bacteria. When the intestinal barrier is compromised, LPS enters the bloodstream, leading to increased production of ROS by NADPH oxidase through activation of the Toll-like receptor 4 (TLR4)-mediated oxidative pathway and resulting in decreased endothelial NO bioavailability due to reduced levels of phosphorylated eNOS, resulting endothelial dysfunction.^12^ A study performed in aging mice found that treatment with exogenous tetrahydrobiopterin increased endothelium-dependent vasodilation and reduced eNOS-derived superoxide formation in the mesenteric arteries, confirming the important role of eNOS decoupling in age-related endothelial dysfunction.^13^

In addition to age-related endothelial dysfunction, oxidative stress also induces cardiomyocyte apoptosis and promotes the development of age-related cardiovascular disease. Loss of cardiomyocytes is a hallmark of heart aging, and can lead to replacement of myocardium and, ultimately, cardiac hypertrophy. ROS was found to induce cardiomyocyte apoptosis through the ERK1/2 MAPK, JNK, P38 MAPK, and CAMKII pathways.^14,15^ Enhanced phosphorylation of JNK and P38 MAPK can promote nuclear translocation of NF-κB, induce cell senescence, and aggravate myocardial damage, while decreased phosphorylation of JNK and P38 MAPK can protect the aging rat heart from oxidative stress.^16^ Some animal experiments have also confirmed the role of oxidative stress in cardiomyocyte apoptosis. Mice with mitochondrial DNA (mtDNA) mutations exhibit an early aging phenotype. In mtDNA mutant mice, oxidative respiratory chains were inhibited, ATP production decreased, ROS accumulation increased, cardiomyocyte apoptosis increased, premature age-related changes in heart tissue were observed, cardiac hypertrophy decreased, and systolic and diastolic function were inhibited.^17^ In conclusion, changes in gut microbiota promote oxidative stress and thus affect the pathogenesis of age-related cardiovascular diseases.

### Mitochondrial dysfunction

**Figure 2.**
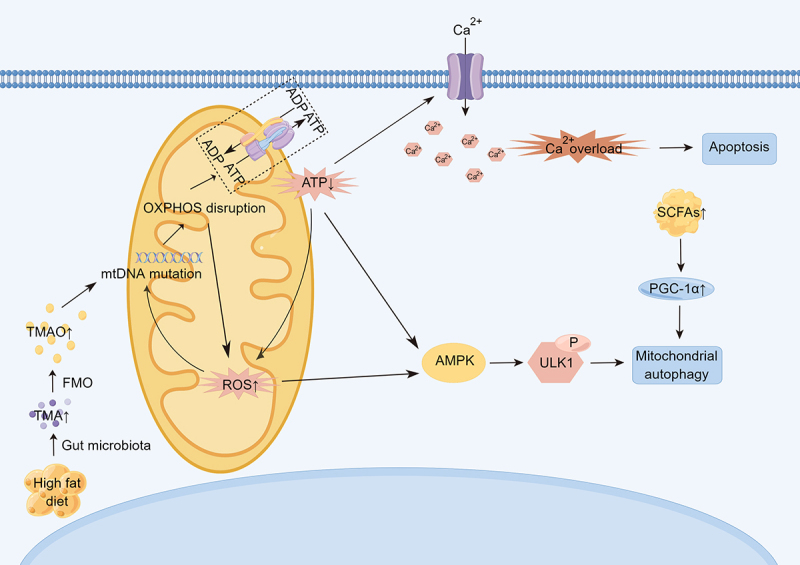
Mitochondrial dysfunction affects the underlying mechanisms of age-related cardiovascular disease. TMAO can promote mitochondrial DNA mutation, thus affecting OXPHOS, increasing ROS production, decreasing ATP/ADP ratio, further damaging mitochondrial DNA, and causing impaired calcium ion metabolism, intracellular calcium overload, and promoting cell apoptosis. Increased oxidative stress activates AMPK, which activates autophagy and degrades damaged mitochondria. (TMAO: trimethylamine N-oxide. OXPHOS: oxidative phosphorylation. AMPK: AMP-activated protein kinase).

Mitochondrial dysfunction is one of the most important mechanisms of age-related cardiovascular disease. In recent years, the relationship among intestinal flora, mitochondrial dysfunction, and age-related cardiovascular disease has attracted wide attention. Intestinal flora metabolites seem to directly affect mitochondrial oxidative stress and formation of the mitochondrial autophagic lysosome, thus regulating inflammatory body activation and inflammatory cytokine production, which are major components of myocardial cell metabolism disorders.^18^ A high-fat diet can impair mitochondrial activity in the colonic epithelium and increase choline metabolism in *Escherichia coli*, thereby increasing circulating TMAO levels and inducing low-grade mucosal inflammation, which plays a key role in age-related cardiovascular disease, especially atherosclerosis.^19^ The microbiota-derived metabolite trimethyl-5-aminovaleric acid was found to reduce fatty acid oxidation (FAO) and affect mitochondrial optic atrophy protein 1 (OPA1) cleavage and oligomerization, thereby severely impairing mitochondrial function and accelerating cardiomyocyte hypertrophy, resulting in functional impairment.^20^ Damaged mitochondria not only produce less ATP, but also release more ROS and have a higher propensity to induce apoptosis, all features of age-related cardiovascular disease.^21^ The mtDNA is located very close to the cellular site of ROS production and lacks protective histones. In addition, the mtDNA repair system is inefficient and prone to mutation.^22^ Mice with mitochondrial mutations, namely early-aging mouse models expressing the sequencing-defective DNA polymerase gamma, exhibited cumulative mtDNA mutations, a significantly accelerated aging phenotype, and an increased risk of heart disease.^23,24^ Wang et al. showed that administration of spermidine (a major mammalian polyamine) to older rats preserved myocardial ultrastructure and reduced cardiac aging by activating mitochondrial biogenesis and inhibiting mitochondrial dysfunction^25^ (Figure 2).

Mitochondrial autophagy, a mitochondrial quality control system, is also believed to be closely related to cardiovascular aging. Mitochondrial autophagy is a selective form of autophagy that degrades old or damaged mitochondria.^26^ Several specific proteins play key roles in the molecular regulation of mitochondrial autophagy, including PTEN-induced kinase 1 (PINK1).^27^ Overexpression of PINK1 has been shown to improve mitosis, thereby protecting vascular smooth muscle cells, while PINK1 deficiency leads to mitotic errors and reduced VSMC survival.^28^ SCFAs produced by intestinal flora can act as signaling molecules to regulate autophagy. When PINK1 was overexpressed in rats treated with rifaximin (an unabsorbable antibiotic that can remodel the intestinal microbiome), the microbial communities producing acetate and propionate and circulating SCFAs levels increased, and PGC-1α expression was activated. PGC-1α acts downstream of SCFAs and is an upstream activator of mitochondrial autophagy and mitochondrial tumorigenesis.^29^

The negative effects of mitochondrial dysfunction on age-related cardiovascular disease provide insight into the cardiovascular aging process and suggest new avenues for developing treatment strategies.

### Inflammation

Inflammation is part of the body’s normal healing and repair response and is essential for protecting the body from bacterial and viral infections, as well as harmful environmental factors.^30^ However, when complex, acute inflammatory responses are not fully resolved, more defense components are mobilized, resulting in a persistent immune response called chronic inflammation.^31^ The gut flora may influence the development of age-related cardiovascular disease by participating in inflammation. An imbalance in the intestinal microecology increases the permeability of the intestinal mucosal barrier, allowing intestinal bacteria and their metabolites to enter the blood circulation, leading to chronic inflammation. Disruption of the intestinal microecology is frequently observed in diseases whose prevalence increases with age, such as obesity and type 2 diabetes.^32^ LPS is thought to be a key factor in this process, as it has a variety of adverse effects on the intestinal tract. For example, it can disrupt intestinal tight junctions via specific signaling pathways, directly causing shedding of intestinal epithelial cells without reclosure of the decompensated tight junction, and may induce oxidative stress, mitochondrial autophagy, and mitochondrial failure in intestinal epithelial cells.^33,34^ In addition, it binds to toll-like receptor 4 (TLR-4) on immune cells to activate pro-inflammatory cascades in local and distant parts of the intestine.^35^ Inflammatory factors such as IL-6, IL-1β, TNF-α, and more characterize the senescence-associated secretory phenotype (SASP).^36,37^ Activation of NF-κB, a key transcription factor involved in the inflammatory response, is thought to play a key role in age-related vascular inflammation, and increased NF-κB expression has been observed in vascular endothelial cells from elderly individuals.^38,39^ Csisza et al. found that NF-κB activation increases monocyte adhesion to endothelial cells in aging arteries, thus making arteries prone to atherosclerosis.^40^ Age-dependent NF-κB activation is also associated with systemic inflammation and impairs endothelium-dependent vasodilation.^41^ Therefore, NF-κB may be a therapeutic target for senile cardiovascular disease in the future. Therefore, it is speculated that TNF-α may play an important role in age-related cardiovascular disease through inflammation mediated endothelial dysfunction.

### Autophagy

Autophagy plays a crucial role in the degradation of long-lived proteins and organelles, and is important for maintaining heart tissue homeostasis during aging. Autophagy appears to decrease with age, and studies in C. elegans, rodents, and human cells have shown an age-dependent decline in lysosomal proteolytic function, which impairs autophagy flux, exacerbates cell damage and leads to the development of age-related diseases.^42,43^ Altered gut microbiota diversity and weakened intestinal barrier integrity can induce abnormal leakage of LPS, and the LPS-activated TLR4 signaling pathway stimulates autophagy in muscle cells, suggesting that LPS can induce excessive autophagy.^44^ In addition, the rate of colonic epithelium autophagy in germ-free mice is lower than that seen in conventionally-fed mice, suggesting that the gut microbiota affects intestinal autophagy under physiological conditions.^45^ *Bifidobacterium dentium* was also found to significantly up-regulate the autophagy signaling pathway and mucin gene expression, enhance intestinal mucous layer and goblet cell function, and net increase mucin production.^46^ This evidence suggests that the gut microbiota participates in autophagy-related processes. The autophagy-related protein Atg5 regulates autophagy and apoptosis. Eisenberg et al. found that mice lacking Atg5 had increased left ventricular hypertrophy and decreased diastolic function, leading to age-related cardiac deterioration.^47^ Beclin-1 is a key factor in autophagosome formation. Hamacher-Brady et al. demonstrated that enhancing autophagy through overexpression of Beclin-1 can actually protect myocardial cells from ischemia-reperfusion injury, while decreasing Beclin-1 expression reduces rational cardiac remodeling.^48,49^ Bcl-2 family proteins can inhibit Beclin-1‘s ability to promote autophagy after binding with Beclin-1. Disruption of the Beclin1-Bcl2 complex has been found to increase autophagy, inhibit age-induced apoptosis, cardiac hypertrophy and fibrosis, and delay cardiac aging.^50^ It is widely believed that autophagy levels decline with age, and in cardiomyocytes this decline leads to the accumulation of dysfunctional organelles and toxic proteins that mediate general cardiac dysfunction. However, others have suggested that it is not that autophagy levels decline, but rather that the level of autophagy activity cannot compete with increasing levels of ROS and oxidative damage.^51^ In addition, decreased autophagy leads to protein degradation and misfolded or damaged protein processing imbalances, which are associated with aging and the occurrence and development of heart disease. Overactivation of autophagy can lead to the degradation of contractile proteins and to cardiomyocyte autophagy.^52^ Therefore, dysregulation of autophagy plays a very important role in age-related cardiovascular diseases.

## Gut microbiota and aging

**Figure 3.**
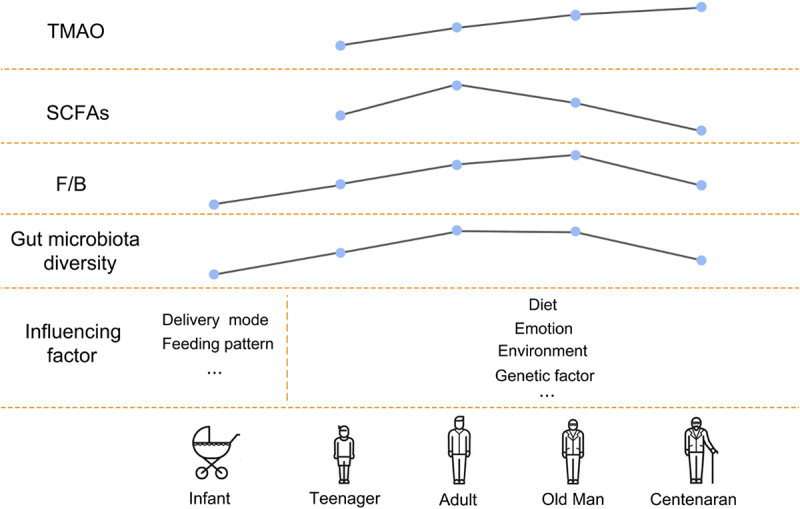
Gut microbiota and aging. The composition of intestinal flora and the level of derived metabolites changed with age. The composition and function of an infant’s early microbiota are influenced by factors such as birth mode and early feeding pattern, and the gut microbiota is influenced by more factors such as genetics, diet, mood, and life events as the infant ages. However, the general trend was that with the increase of age, the diversity of intestinal flora increased, the number of *Bacteroides* decreased significantly, and the number of *Firmicutes* increased, the level of TMAO increased, and the abundance of SCFAs producing bacteria decreased. However, due to the existence of higher inflammation levels in centenarians, the diversity of intestinal flora decreased, the proportion of *Firmicutes* decreased, and *Bacteroides* increased. (TMAO: trimethylamine N-oxide. SCFAs: short-chain fatty acids).

Genetics and environment interact in complex ways to influence aging. The gut microbiome changes dramatically throughout an individual’s life. The composition and function of an infant’s early microbiota are mainly determined by delivery method and early feeding, followed by the maternal microbiota and antibiotic exposure.^53^ *Lactobacillus* and *Bifidobacterium* predominate in breastfed infants, while *Enterococcus*, *Bacteroides*, *Streptococcus*, *Clostridium* and *Enterobacter* predominate in formula-fed infants.^54^ In addition, Dominguez-Bello et al. showed that the skin surface bacteria in infants born by C-section mainly comprises *Staphylococcus*, *Corynebacterium*, and *Propionibacterium*; while babies delivered vaginally have the same skin bacterial community as their mothers, even though their mothers’ vaginal microbiomes largely consist of *Lactobacillus*, *Prevotella*, or *Cilium*.^55^ The diversity of the human microbiome increases with age and plateaus in adulthood. Roswall et al. showed that the microbiota of infants at 4 and 12 months is highly heterogeneous and very different from that of adults, but that the microbiota of children at 3 and 5 years is more similar to that of adults.^56^ As people age, their gut microbiota is influenced by a variety of factors, such as genetics, diet, mood, and life events. However, in general *Bacteroides* dominates the gut microbiota in children and decreases significantly with age, while *Firmicutes* increases.^57^ Middle-aged adults and elderly individuals exhibit similar gut microbiota structure, i.e., *Bacteroides* and *Firmicutes* are dominant (about 95% of the microbiota), and *Actinobacteria* and *Proteus* are present in smaller proportions.^58^ However, centenarians stand out as a separate population with a microbiome that displays distinctive traits and has low diversity in terms of species composition. *Bacteroides* and *Firmicutes* remain dominant in the gut microbiota of centenarians (accounting for more than 93% of the total bacteria), but a decreased proportion of *Firmicutes* and an increased proportion of *Bacteroides* were observed compared with younger adults, and the gut was rich in *Proteus*.^58,59^ This change that is thought to be likely to lead to a compromised immune system, which in turn is associated with more inflammation^58^ (Figure 3).

Age and renal function have significant effects on intestinal microbial composition and the levels of metabolites derived from intestinal flora. The intestinal flora of the elderly is characterized by a decrease in glycolysis genes and an increase in proteolytic genes, thus promoting the excessive growth of pathogenic bacteria and aggravating the inflammatory response. Inflammation is accompanied by massive ROS production, which inactivates strict anaerobic bacteria (i.e., *Firmicutes*) and promotes facultative aerobe,^60^ The increased expression of inflammatory cytokines reduces the expression of tight junction proteins. This further increases intestinal permeability and may perpetuate inflammation.^61,62^ With age and deterioration of renal function, TMAO level increases,^63^ the abundance of SCFA-producing bacteria decreases,^64^ and bile acid composition becomes unbalanced.^65^ Studies have shown that patients with chronic kidney disease (CKD) have reduced intestinal bacterial α diversity, altered community composition, and significantly higher TMAO levels than patients with other diseases.^66^ Exogenous choline supplementation or increased dietary intake of TMAO can lead to the development of renal tubulointerstitial fibrosis and early renal dysfunction.^67^ Exogenous butyrate supplementation improved AMPK phosphorylation in a rat model of CKD, increased glucagon-like peptide-1 secretion, and promoted colonic mucin and tight junction protein expression levels, thereby strengthening the intestinal barrier and reducing LPS leakage and inflammation.^68^ Treating diabetic mice with a selective agonist of TGR5, a membrane Ba-activated G-protein – coupled receptor whose activation can inhibit inflammation and reduce atherosclerosis, increased mitochondrial biogenesis and fatty acid β oxidation in the kidney and reduced oxidative stress, as well as having certain anti – renal fibrosis effects.^69^ In addition, intestinal homeostasis imbalance increases with age, which leads to increased intestinal microbiota LPS production, Toll-like receptor activation, and mucous layer degradation, further increasing intestinal permeability and accelerating systemic endotoxemia and inflammation.^70,71^ Yamamoto et al. showed that the induction amplitude of CD14 and TLR4 mRNA in the liver and kidney of older mice treated with LPS was greater than that seen in young mice, and that significantly increasing *CD14 and TLR4 gene* expression enhanced LPS binding and intracellular signaling, mediating many inflammatory responses involved in systemic damage.^72^

## Gut microbiota in age-related cardiovascular diseases

### Atherosclerosis

Atherosclerosis (AS) is a chronic metabolic disease caused by abnormal accumulation of lipids and complex carbohydrates in the inner wall of the arteries that leads to artery hardening and the formation of secondary lesions. AS is the pathological basis of coronary heart disease, cerebral infarction, and other serious cardiovascular diseases. It is generally believed that the atherosclerotic plaque itself is a microbial environment. Haraszthy et al. showed that atherosclerotic plaques in human endarterectomy specimens contain DNA from a variety of bacteria, such as *Prevotella intermedia*, *Porphyromonas gingivalis*, *Actinobacillus actinomycetemcomitans*, and *Bacteroides forsythus*.^73^ However, the intestinal microbiota of patients with atherosclerotic cardiovascular disease is different from that of healthy individuals. The intestinal microbiota of patients with atherosclerotic cardiovascular disease has higher levels of *Streptococcus* and *Enterobacteriaceae*,^74^ indicating that alterations in the intestinal microbiota are indeed associated with atherosclerosis.

An imbalance in the intestinal flora affects the development of atherosclerosis, mainly through oxidative stress and inflammation. H. pylori has been found to activate NADPH oxidase, which produces ROS.^8^ REDOX-sensitive signal transduction is activated by NF-κB, activating protein-1 (AP-1), and mitogen activating protein kinase (MAPK), which induces the release of inflammatory cytokines and ultimately vascular inflammation through upregulation of adhesion molecules.^75,76^ Intestinal flora metabolites are also involved in promoting atherosclerosis. In the study by Vienna E. Brunt et al., plasma TMAO levels in mice were found to increase with age and lead to endothelial dysfunction through oxidative stress and inflammation.^77^ As an endogenous ligand, TMAO can bind to TLR to increase the secretion of pro-inflammatory cytokines IL-1β and IL-18 as well as ROS.^78,79^ ROS can damage cells, leading to lipid peroxidation and the oxidation of low-density lipoprotein (LDL). Oxidized low-density lipoprotein (ox-LDL) remains in the intima of blood vessels and induces endothelial cell (EC) dysfunction.^80^ Scavenger receptors expressed on macrophages (mainly scavenger receptor type A (SR-A) and some CD36 type B family members) are key to the ingestion of ox-LDL molecules by macrophages, which induces their transformation into foam cells.^81^ Microbial TMA-producing enzyme inhibitors have been found to inhibit platelet reactivity and the rate of thrombosis in animals, as well as inhibiting foam cell formation and atherosclerosis development and reducing the number of bacteria associated with TMAO production.^82,83^ In addition, LPS and other bacterial components (such as peptidoglycan) can leak into the circulation when intestinal permeability increases, triggering an inflammatory response while stimulating LDL uptake; this reduces cholesterol production by foam cells, promoting monocyte recruitment and macrophage foam cell formation, ultimately leading to atherosclerosis.^84^ SCFAs, which are intestinal microbe metabolites, have been found to be less abundant in the intestinal flora of patients with atherosclerosis than in healthy individuals.^85^ SCFAs have anti-inflammatory effects and can inhibit the NF-κB activity of immune cells and reduce the production of pro-inflammatory cytokines.^86,87^ A study performed in human mononuclear macrophages showed that exogenous SCFA supplementation significantly inhibited NF-κB p65 activation and ROS production, thereby inhibiting activation of the NLRP3 inflammasome and slowing progression of atherosclerotic plaque formation.^88^ Exogenous propionic acid supplementation modulates intestinal Niemann-Pick C1-like protein-1, a major transmembrane transporter that mediates intestinal cholesterol absorption, thereby reducing cholesterol absorption and inhibiting the development of atherosclerosis.^89^ In addition, sodium butyrate can inhibit PI3K/Akt/mTOR signaling pathway mediated autophagy, enhance Atg5-mediated autophagy, reduce α-synaptic nucleoprotein expression, increase human microtubule-associated protein light chain 3II (LC3II) expression, reduce p62 expression, and play a protective role in arteriosclerosis.^90^ Surprisingly, BAs regulate metabolites derived from the gut microbiota, such as TMAO, and play a role in atherosclerosis. BAs are the end product of cholesterol catabolism in the liver. Conversion of cholesterol into BAs is the primary means of eliminating excess cholesterol and maintaining systemic cholesterol homeostasis. In vivo, the farnesol X receptor (FXR) upregulates flavin-containing monooxygenase-3 (FMO3) and increases the plasma TMAO level, thus promoting atherogenesis.^91,92^ TMAO also activates FXR and nuclear receptor small heterodimer chaperone (SHP) by altering the bile acid profile, downregulates cholesterol 7α-hydroxylase (CYP7A1) expression, and restricts BA synthesis in the liver, thus accelerating the formation of aortic lesions in Apolipoprotein E knockout (ApoE^−/−^) mice.^93^ Finasteride, a specific inhibitor of intracellular enzyme-type II 5A-reductase, has been shown to improve gut microbiota richness and diversity, thereby downregulating FMO3 expression, which reduces circulating TMAO levels and inhibits the development of atherosclerosis.^94^ Little is known about how the gut microbiome mediates mitochondrial dysfunction and thus plays a role in atherosclerosis. However, there is some evidence that changes in intestinal microecology after Canagliflozin treatment can improve cardiac mitochondrial balance, relieve oxidative stress, reduce lipid accumulation, and reduce plasma arteriosclerotic index and atherogenic index.^95^ This has led to the hypothesis that gut flora is mitochondrial dysfunction – mediated atherosclerosis. In addition to TMAO, LPS and SCFAs, BAs, indole sulfate, a tryptophan metabolite derived from the gut microbiota, has been shown to play a role in AS by promoting pro-inflammatory macrophage activation and thus vascular inflammation,^96^ while indole-3-propionic acid (IPA) has a protective effect. IPA promotes the reverse transport of cholesterol by macrophages and alleviates the development of atherosclerotic plaques in ApoE^−/−^ mice.^97^ Gut microbiota – derived phenylalanine metabolite phenylacetylglutamine (PAGln) accelerates platelet clot formation and enhances thrombosis potential.^98^ For example, greater numbers of microbes associated with PAGln synthesis, such as *Roseburia*, *Blautia*, and *Ruminococcus*, as well as elevated plasma levels of intestinal microbiota – derived metabolite PAGln, were detected in CAD patients with in-stent restenosis.^99^

### Hypertension

**Figure 4.**
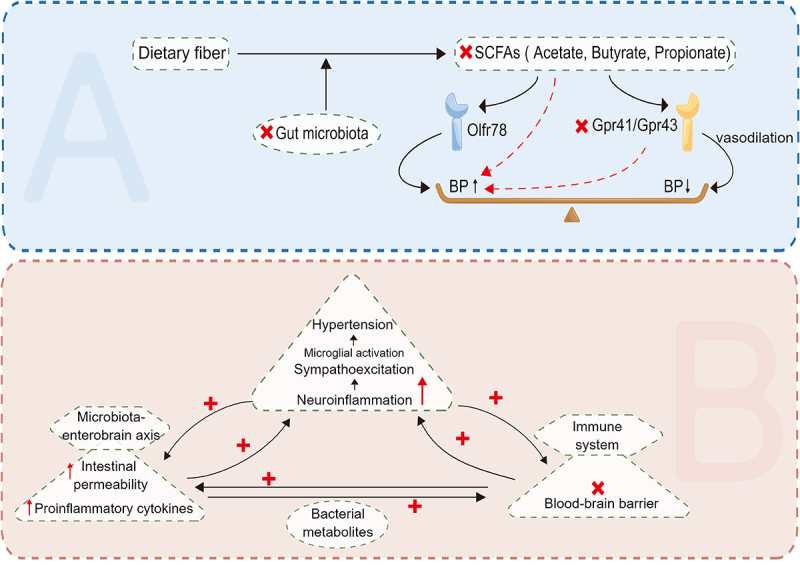
The relationship between hypertension and SCFAs and neuroinflammation. SCFAs increases blood pressure through Olfr78, and also acts on Gpr41 and Gpr43, promoting vasodilation and thus lowering blood pressure. At the same time, neuroinflammation and activity imbalance of autonomic nervous system caused by gut-brain axis dysfunction can also lead to elevated blood pressure. (SCFAs: short-chain fatty acids).

Due to a combination of genetic and environmental variables, hypertension is one of the most common chronic diseases and a significant risk factor for cardiovascular and kidney disease. Intestinal ecological imbalance may be a potentially modifiable risk factor for hypertension.^100^ The microbial-gut-brain axis refers to bidirectional communication between the gut microbiota and the brain mediated by humoral factors such as TMAO and other metabolites (Figure 4). Changes in this axis are believed to be related to blood pressure regulation.^101^ It is thought that dysfunction of the gut-brain axis can lead to elevated levels of inflammatory mediators, intestinal flora metabolites, circulating bacteria, and other factors in the circulation that cause neuroinflammation and disrupt autonomic nervous system activity, thus increasing blood pressure, which in turn has a negative impact on intestinal function, resulting in a vicious cycle.^102^ Neuroinflammation can promote hypertension by enhancing sympathetic nervous system activity.^102^ Long-term high-salt diets have been shown to increase TMAO levels in the blood and brain, promote neuroinflammation and oxidative stress in the paravicular nucleus of the hypothalamus, and increase the production of pro-inflammatory cytokines such as IL-1β, TNF-α, and NF-κB in the brain, leading to increased sympathetic activity and the development of hypertension.^103^ Treating rats with DMB (an inhibitor of TMAO formation) reduces neuroinflammation and oxidative stress in the hypothalamic paraventricular nucleus, thereby improving high-fat diet – induced sympathetic excitation and hypertension.^103^ As mentioned above, TAMO has a significant effect on kidney function. Circulating TMAO not only causes kidney damage by increasing blood pressure, but also directly promotes renal interstitial fibrosis and dysfunction by mediating TGF-β/Smad3 phosphorylation.^104^ In patients with end-stage renal disease due to the accumulation of circulating metabolic waste, intestinal cell damage is increased, intestinal barrier function is impaired, and local inflammation and cytokine production are triggered, thus exacerbating adverse cardiovascular outcomes and mortality.^104,105^ In addition, SCFAs have been found to regulate blood pressure through Olfr78，Gpr41，and Gpr43. SCFAs act on renal afferent arteries through Olfr78 to increase renin release and promote basal renin levels, thereby increasing blood pressure.^106^ SCFAs also act on Gpr41 and Gpr43 to promote vasodilation and thus reduce blood pressure.^107^

Neuroinflammation can also disrupt the blood-brain barrier and affect the development of hypertension. Disruption of the blood-brain barrier may allow circulating inflammatory factors or intestinal metabolites to enter the brain, thereby affecting microglia function. LPS can be recognized by pattern recognition receptors (PRRS) on microglia cell membranes, activate local immune responses and increase levels of neuroinflammation, thus having a hypertensive effect.^101^ In addition, microglia are activated by hypertensive stimuli (such as high-salt diet, Ang II, etc.) and release pro-inflammatory cytokines such as TNF-α, IL-1β, and IL-6 and anti-inflammatory cytokines such as IL-10, while inhibition of Ang II signaling reduces neuroinflammation and restores blood pressure.^108,109^ In a study of 60 diabetic patients, Roshanravan et al. found that exogenous sodium butyrate and inulin supplementation can significantly reduce TNF-α mRNA levels, which may affect the Ang II signaling pathway and thus significantly reduce diastolic blood pressure.^110^ At the same time, minocycline attenuates microglia activation, inhibits neuroinflammatory processes and rebalances the gut microbiome to play an antihypertensive role.^111^ In summary, an imbalance in the intestinal flora can cause neuroinflammation, thus promoting hypertension. Therefore, it seems likely that addressing intestinal flora imbalance with targeted drugs could inhibit neuroinflammation and thus control blood pressure.

### Myocardial infarction and heart failure

Myocardial infarction is one of the most common causes of heart failure and cardiogenic death worldwide. While percutaneous coronary intervention (PCI), also known as mechanical reperfusion, has reduced acute mortality from myocardial infarction, the incidence of cardiovascular events after myocardial infarction remains high.

Increased intestinal permeability and bloodstream infection are typical complications associated with myocardial infarction and heart failure.^112^ *Lactobacillus, Bacteroides*, and *Streptococcus*, possibly from the gut, have been detected in the blood microbiome of patients with acute ST-segment elevation myocardial infarction.^113^ Reduced intestinal blood flow, increased gastrointestinal symptoms, increased serum LPS and elevated IgA-anti-LPS levels were also found in patients with chronic cardiac dysfunction.^114,115^ It has been suggested that the accumulation of intestinal bacteria and intestinal LPS in the systemic circulation triggers the recruitment of monocytes, which activate systemic inflammation and ultimately lead to cardiovascular events after myocardial infarction.^113^ LPS plays an important role in the progression of coronary heart disease by initiating the transcription of inflammatory factors, inducing the release of inflammatory factors, and inducing chronic low-grade inflammation through the TLR4/MyD88/NF-κB pathway.^116^ Polymyxin B was used in mice for three consecutive days to inhibit intestinal bacterial transfer and eliminate microbial products from systemic circulation, and significant reductions in plasma LPS levels, myocardial infarction size, local inflammation, and myocardial fibrosis were observed.^113^

SCFAs secreted by intestinal flora are usually absorbed by epithelial cells. They act on mitochondria and stabilize hypoxia-inducing factors. Intestinal hypoxia caused by visceral under perfusion leads to intestinal acidification and a decrease in the number of butyrate-producing bacteria.^117^ In addition, one study found that dietary supplementation with SCFAs or monocyte transplantation after myocardial infarction can significantly reverse the adverse effects of antibiotics on mortality and ventricular rupture rate,^118^ suggesting that SCFAs is beneficial for cardiac repair and improves prognosis after myocardial infarction.

Some studies have found that primary BA levels are increased and secondary BA levels are decreased in patients with chronic heart failure.^119^ Given that secondary BAs are generated from primary BAs by intestinal microbes,^120^ this suggests that intestinal microbes are related to heart failure. As mentioned earlier, PAGln plays a role in the formation of blood clots, and it has also been found to play an important role in heart failure. An important manifestation of heart failure is sympathetic overactivation. Although sympathetic overactivation can maintain ejection fraction in the short term, decompensated heart failure will still occur in the long term. PAGln can aggravate heart failure by activating G-protein – coupled receptors such as the α2A, α2B, and β2 adrenergic receptors,^98^ to a certain extent causing overactivation of the sympathetic nervous system.^121^ Furthermore, it has been demonstrated that increasing PAGln concentrations correlate with increasing heart failure severity.^122^ This suggests that PAGln may be a potential predictor of heart failure severity. In addition, the gut microbiota that produce imidazole propionate (ImP) such as *Streptococcus mutans, Clostridium symbiotic* and *Eubacterium eligen* have also been found to be enriched in patients with heart failure.^123^ ImP impacts insulin signaling at the insulin receptor substrate level by activating p38γ MAPK, thereby promoting p62 phosphorylation and subsequently inducing rapamycin complex 1 (mTORC1) signaling; these mechanisms are associated with cardiac fibrosis, hypertrophy, and heart failure.^124^

### Myocardial hypertrophy and fibrosis

Pathological cardiac remodeling, characterized by heart hypertrophy and fibrosis, is a pathological feature of many heart diseases and can lead to heart failure and cardiac arrest. Although few studies have been published on the relationship between the intestinal flora and myocardial hypertrophy and fibrosis, there is some evidence that there is a relationship between the two. One study showed that administering probiotics, prebiotics, and biotin to improve the composition and function of the intestinal flora in rats significantly reduced the cardiac hypertrophy caused by long-term exposure to hypobaric hypoxia.^125^ This suggests that a normal gut flora is essential to prevent cardiac hypertrophy and fibrosis. Asgharzadeh et al. showed that LPS reduces cardiac antioxidant levels, increases cardiac oxidative stress, and induces myocardial and perivascular fibrosis in rats.^126^ Singh’s experiments in mice confirmed that LPS also activated TLR-4, thereby inducing the activation of CaMKII in cardiomyocytes. As a signaling molecule for myocardial infarction and ROS activation, overexpression of heart-specific transgenic CaMKII has also been found to cause myocardial hypertrophy, heart failure, and premature death.^127^ In addition, it has been found that elevated circulatory TMAO levels increase the likelihood of developing heart failure, as they are associated with pathological dilatation of the left ventricle, reduced left ventricular ejection fraction, increased circulating BNP levels, and increased pulmonary edema and myocardial fibrosis^128^. Li et al. demonstrated that TMAO at least partially aggravates doxorubicin-induced myocardial fibrosis in mice by activating NLRP3 inflammasomes.^129^ As previously mentioned, NF-κB activates the inflammatory response, and NF-κB and Smad3 play important roles in myocardial fibrosis. TMAO promotes cardiac and renal interstitial fibrosis by activating NF-κB and Smad3. TMAO inhibition reduces NF-κB and Smad3 activation to prevent cardiac remodeling and renal interstitial fibrosis in a variety of heart and kidney diseases.^130^ For example, Organ et al. found that the microbial enzyme choline TMA lyase blocked the conversion of choline to TMAO at the gut microbiota level, significantly improved left ventricular remodeling, and significantly reduced left ventricular pro-fibrotic gene expression.^131^ The intestinal flora has been shown to regulate key transcription factors, coactivators, and enzymes related to mitochondrial biogenesis and metabolism, and can directly affect mitochondrial oxidative stress and the formation of mitochondrial autophagic lysosomes, thereby regulating the activation of inflammatory bodies and the production of inflammatory cytokines, which are major factors in myocardial or cardiomyocyte metabolic disorders.^18^ Pyridine is an acetylcholinesterase inhibitor that inhibits intestinal inflammation, restores intestinal microbiome homeostasis, reduces the abundance of microorganisms that produce branched amino acids, and improves intestinal barrier integrity to decrease circulating levels of branched amino acid.^132,133^ Yang et al. showed that pyridostigmine alleviated mitochondrial structural abnormalities in mice with diabetic cardiomyopathy induced by a high-fat diet plus streptomycin, increased ATP production, reduced reactive oxygen generation, reduced mitochondria-related cell apoptosis, and alleviated cardiac insufficiency, hypertrophy, and fibrosis.^133^

### Arrhythmia

Arrhythmias are caused by abnormal excitation of the SA node or by excitation generated outside the SA node. Excitation conduction is slow, blocked, or conducted through abnormal channels, with the origin of heart activity and/or conduction disorders resulting in abnormal heart beat frequency and/or rhythm. Recent studies have found that several risk factors for atrial fibrillation, such as diabetes mellitus, obesity, hypertension, obstructive sleep apnea, and coronary artery disease, are independently associated with the progression of atrial fibrillation, as well as with intestinal ecological disorders.^134^ In other words, arrhythmias and intestinal disorders may share some of the same risk factors and are closely related. Similar to other cardiovascular diseases, butyrate-producing bacteria such as *Faecalibacterium* and *Oscillibacter* have decreased abundance in the gut of patients with atrial fibrillation, while *Ruminococcus*, *Streptococcus*, and *Enterococcus* are significantly increased in the gut of patients with atrial fibrillation.^135^ One study found that the levels of *Bacteroides* and *Prevotella* in a high-risk CHA2DS2-VASc group were higher and lower, respectively, than those in a low-risk CHA2DS2-VASc group, suggesting that the risk of thromboembolism in patients with atrial fibrillation is associated with intestinal flora.^136^

The relationship between atrial fibrillation and inflammation, especially the NLRP3 inflammasome, which is involved in the pathogenesis of atrial fibrillation, has recently attracted increasing attention. Right atrial cardiomyocytes showed higher NLRP3 inflammasome activity in patients with paroxysmal and chronic atrial fibrillation compared with the control group (which comprised individuals with a normal sinus rhythm).^137^ A selective inflammasome inhibitor (MCC950) blocked AF induction by preventing NLRP3 inflammatory complex formation. Furthermore, SCFAs attenuate NLRP3 inflammasome activation through the GPR43 signaling pathway and ultimately induce NLRP3 degradation through the autophagy pathway, thereby reducing the occurrence of atrial fibrillation.^138^ Mice with low SCFAs levels have an increased vulnerability to atrial fibrillation, which can be alleviated by SCFAs supplementation.^139^ In addition, high TMAO levels promote atrial fibrillation by inducing left atrial inflammation and fibrosis;^140^ indeed, injection of TMAO into the atrial autonomic ganglion cluster in a canine model increased local expression of pro-inflammatory cytokines. While elevated TMAO levels appear to be associated with increased nerve plexus activity, shortening of the atrial effective refractory period due to atrial tachycardia, and increased susceptibility to atrial fibrillation,^141^ more research is needed to confirm these findings. TMAO also induces a significant upregulation in the expression of pro-inflammatory markers such as IL-1β, IL-6, and TNF-α in tyrosine hydroxylase – positive neurons, which may lead to sympathetic overactivation in the left stellate ganglion and contribute to the occurrence of ventricular arrhythmias.^142^ LPS is also associated with arrhythmia, and administration of LPS in dogs leads to elevated levels of TNF-α and IL-6 in the blood and right atrium, which may play a role in LPS-induced NF-κB activation and increase susceptibility to atrial fibrillation in the context of systemic inflammation through the adrenergic receptor dependent pathway.^143^ In addition to inflammation, oxidative stress also plays an important role in arrhythmia. PAGln has been found to increase apoptosis, ROS production, and CaMKII and ryanodine receptor activation, as well as reduce cell viability. In short, PAGln can promote oxidative stress and atrial cell apoptosis to promote atrial fibrillation.^144^

## Gut microbiome as a potential therapeutic direction

The association of gut microbiota composition and gut microbiota – derived metabolites with age-related cardiovascular disease suggests that the gut microbiota may be a potential therapeutic target for age-related cardiovascular disease. Improving gut microbial diversity and depleting or supplementing gut microbiota – derived metabolites with synthetic analogs are two broad categories of potential intervention that are currently under investigation.^145^ FMT, which involves the transfer of stool from a healthy donor to the gut of another patient, can reverse the recipient’s intestinal microbiome imbalance to a certain extent.^1^ Studies have found that the relative abundance of *Bacteroides* in the stool of patients receiving FMT increases, while the abundance of Proteus decreases; in addition, the fecal microbiome of recipients is more diverse after transplantation compared with before transplantation.^146^ A clinical study of 18 treatment-naive male patients with cardiometabolic syndrome who received a single duodenal infusion of allogeneic FMT or autologous FMT from a lean, healthy donor showed improvement in peripheral blood insulin sensitivity 6 weeks after the infusion. In addition, bacterial diversity and the number of butyrate-producing bacteria increased.^147^ At present, there are few clinical reports on the effect of FMT on age-related cardiovascular disease, but intestinal flora diversity changes with age, so targeted FMT treatment for different age groups is a potential treatment. In addition, the beneficial effects of FMT on intestinal flora diversity only last for a few weeks,^148^ so further research is needed to determine how to maintain the effects of this treatment.

As described above, FXR plays a key role in TMAO production and cholesterol metabolism. FXR is distributed in the liver, small intestine, pancreas, kidney, adrenal gland, and other parts of the human body. It plays a role in regulating bile acid metabolism, lipid metabolism, and glucose metabolism, and has been shown to play a protective role in nonalcoholic fatty liver disease^149^ and acute kidney injury.^150^ Therefore, specific inhibition of FXR expression in the intestine could reduce TMAO production without affecting cholesterol metabolism, and as such is a promising therapeutic approach.

## Conclusion

Despite the many clinical treatments available for cardiovascular disease, cardiovascular disease remains the leading cause of death worldwide. The gut microbiota is strongly associated with cardiovascular diseases, which provides potential new therapeutic targets for treating age-related cardiovascular disease. Maintaining a healthy gut flora is essential, not only for proper food digestion and absorption, but also for proper intra-organ metabolism. Especially for patients with cardiovascular disease, dietary optimization is important for maintaining good health. Prebiotics can have beneficial effects on host health by selectively stimulating the growth and/or activity of a healthy gut microbiota. In particular, targeted supplementation with probiotics reduces the abundance of harmful components of the gut microbiota. Regarding FMT, it remains unclear whether infectious diseases can be transmitted to patients through donated stool, whether adverse reactions occur after transplantation, how long the beneficial effects of the transplantation last, and whether additional transplantations are needed to maintain the effects, and more research is needed to address these issues in the future. The gut microbiome may an important target of precision medicine, and increasing our understanding of the underlying mechanisms by which the gut microbiota affects cardiovascular health could help improve the health of patients with age-related cardiovascular disease in the future.

## Data Availability

The data used to support the findings of this study are included within the article.
